# Disappearing Inferior Vena Cava in A Pediatric Patient with Down Syndrome and Hereditary Thrombophilia

**DOI:** 10.5334/jbr-btr.975

**Published:** 2016-02-08

**Authors:** Bulent Petik, Muhammer Ozgur Cevik, Mehmet Sirik, Deniz Colak, Sukru Mehmet Erturk

**Affiliations:** 1 Radiologist, Department of Radiology, Adiyaman University Medical Faculty, Adiyaman, Turkey; 2 Medical Genetic Specialist, Department of Medical Genetics, Adiyaman University Medical Faculty, Adiyaman, Turkey

**Keywords:** Disappearing Inferior Vena Cava, Hereditary Thrombophilia, IVC, Down syndrome, homozygous MTHFR polymorphism

## Abstract

Absence of the infrarenal segment of the inferior vena cava is an extremely rare anomaly. The reasons for such a developmental failure are unclear. Most researchers believe that the cause lies in embryonic dysgenesis affecting separate segments or the entire inferior vena cava. Others suggest that absence of the inferior vena cava is not embryonic in origin, rather the result of intrauterine or perinatal thrombosis. We report a case here that during a period of six months, inferior vena cava first occluded, then become redundant in a baby girl with several chromosomal and gene defects, including Down syndrome and hereditary thrombophilia, admitted to our hospital due to the swelling and redness of the right lower extremity. From this observation, we propose that the absence of the inferior vena cave was not of embryonic origin but due to thrombosis.

## Introduction

The embryogenesis of the inferior vena cava (IVC) is a complicated process. Although there are numerous IVC anomalies and variations, the rarest and most severe one among them is the absence of the IVC [1,2,3]. This absence can be either complete or complete with preservation of the suprarenal segment [1,2,3,4]. Some authors proposed that the agenesis of IVC is not a congenital disease, but caused by intrauterine or perinatal thrombosis of the IVC [1,4,5,6].

We present a 10 month old baby girl who had a *disappearing* IVC with preservation of suprarenal segment, generalized thrombosis in the right lower extremity and in the bilateral common iliac veins. In the follow-up imaging studies performed six months later, the suprarenal segment was thrombosed, as well. The patient had Down syndrome (trisomy 21) and pericentric inversion of ninth chromosome, a condition which is generally accepted as a harmless polymorphism. Nevertheless, the patient had hereditary thrombophilia due to her homozygous MTHFR (A 1298C) polymorphism, HPA 1 (b/b), factor V Leiden (G1691A) heterozygous and ACE I/D genotype (Del/Del).

## Case report

A ten-months-old girl was referred to our clinic with swelling and warmth and red discoloration in the right lower extremity. Regarding the clinical history, she had growth retardation and protein-energy malnutrition.

**Figure 1:**
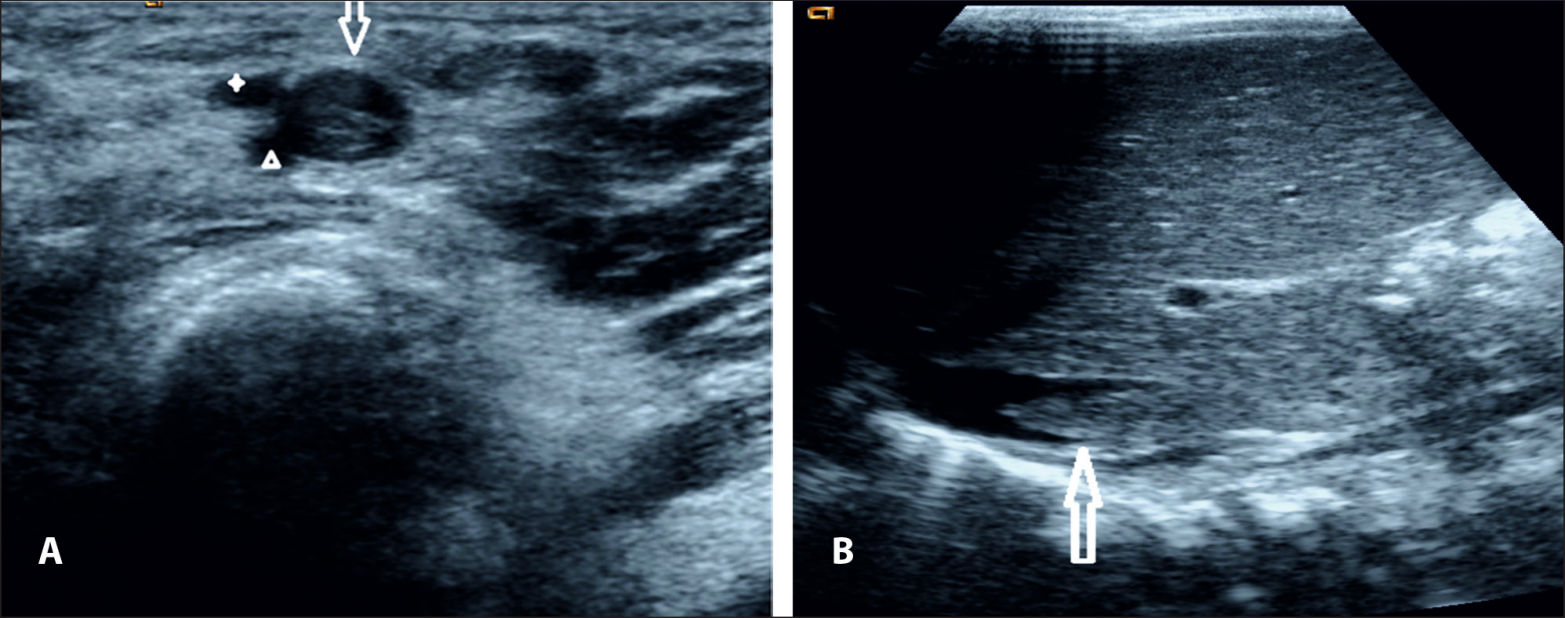
Ultrasound image showing (A) echogenic thrombus (arrow) in the infrarenal IVC and (B) a thrombosed common femoral vein (arrow) adjacent to superficial (asterix) and deep (arrowhead) femoral arteries.

On the color Doppler ultrasound examination, there was generalized thrombus within the great and small saphenous veins, popliteal vein, superficial and deep femoral veins, and common femoral vein. Upon seeing this and noting the visible surface veins both in the right lower extremity and abdomen, an intraabdominal venous color Doppler ultrasound was performed. Bilateral common iliac veins and infrarenal and renal segments of IVC were thrombosed, as well. Suprarenal and intrahepatic segments of IVC were preserved (Figure 1). No family history of deep venous thrombosis or IVC agenesis were noted (neither in her parents nor in her two elder brothers). A detailed genetic testing revealed that the patient has Down syndrome (with one extra twenty-first chromosome) and pericentric inversion in the ninth chromosome that p11 and q13 are inversely translocated (47, XX, inv (9p11q13), +21). Patient’s cardiovascular risk analysis panel revealed that she had increased venous thrombosis risk with homozygous MTHFR (A 1298C) polymorphism, HPA 1 (b/b), factor V Leiden (G1691A) heterozygous and ACE I/D genotype (Del/Del); other panel polymorphisms were normal (MTHFR C677T polymorphism was C677C, factor XIII V34L polymorphism was V34LL, PAI- Serpine1 mutation 4G/5G test was 5G/5G, Prothrombin G20210A was 20210GG, Beta Fibrinogen 455A > G polymorphism was 455G > G, APO B R3500Q polymorphism was 3500RR). In addition, the patient’s mother had a 46, XX, inv (9p11q13) karyotype, which represents the normal female with peripheric inversion at the ninth chromosome. Mother had also increased venous thrombosis risk confirmed by heterozygous PAI-SERPINE1 4G/5G, MTHFR (A1298C) heterozygous, ACEI/D Ins/Del, HPA 1 a/b a/b; another panel polymorphisms were normal. She gave birth to two older boys who were phenotypically normal and were not genetically analyzed. Her father had a normal karyotype 46, XY with increased venous thrombosis risk confirmed by homozygous MTHFR (A1298C), HPA 1 a/b a/b, and ACEI/D Ins/Ins; another panel polymorphisms were normal.

**Figure 2:**
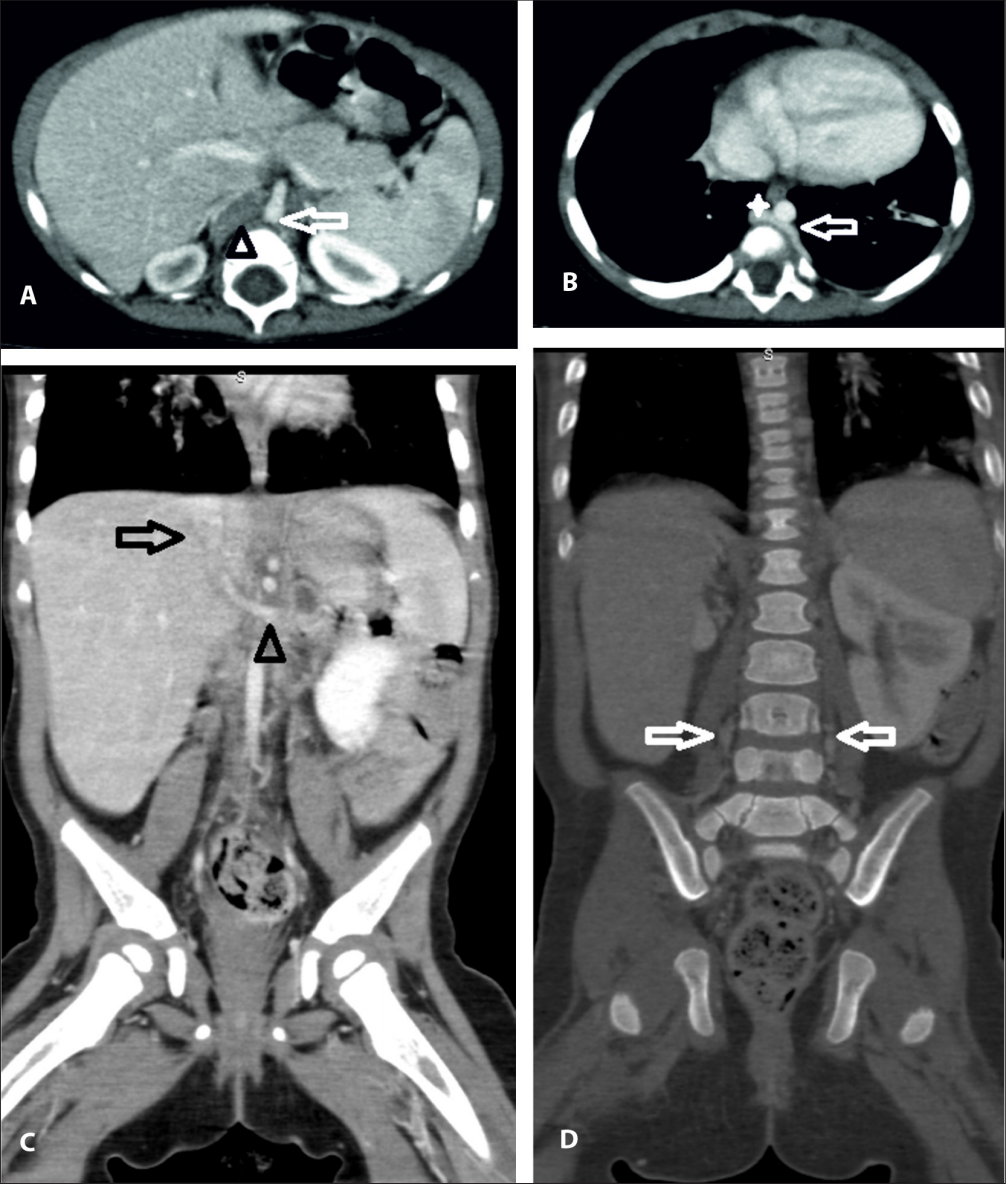
Contrast-enhanced computed tomography, showing in the axial plane (A) hemiazygous vein (arrow) draining into a continuous azygous vein (asterix) and (B) absence of opacification of the inferior vena cava (arrowhead) adjacent to aorta (arrow). Coronal reformation images showed (C) dilated ascending lumbar veins (arrows) and (D) the drainage of the left renal vein (arrowhead) into the intrahepatic segment of IVC (arrow).

After initial visit, the patient was lost to follow-up. Six months later she was referred from the emergency unit with similar symptoms to our clinic. This time, on the color Doppler ultrasonography, in addition to the thrombotic venous structures observed previously, the suprarenal segment of her inferior vena cava (IVC) was also thrombosed. A Computed tomography scan (CT) showed thrombosed (*disappearing*) IVC, the continuous hemiazygous vein draining into the continuous azygous vein, draining of the left renal vein into the intrahepatic segment of IVC, collateral veins in the lower extremities and pelvis, dilated ascending lumbar veins (Figure 2). A detailed physical exam revealed that she developed secondary pulmonary hypertension and cardiac murmur.

## Discussion

We present an interesting case with generalized thrombosis of IVC (the only hepatic segment was preserved) and Down syndrome together with a pericentric inversion of ninth chromosome that is usually referred as harmless polymorphism. Both of her parents had increased risk for venous thrombosis due to their polymorphisms in selected genes responsible for hemodynamic properties of blood circulation.

Down syndrome appears to have a relationship with autoimmune disorders, including Moyamoya and Graves’ diseases that are associated with antithyroid microsomal antibodies and antiphospholipid antibodies [7,8,9] and antiphospholipid antibodies are related to an increased risk of thrombotic events generally. Furthermore, there are some reported cases with an association of superior sagittal sinus thrombosis and trisomy 21 [10,11]. Nevertheless, in the present case, we believe that the thrombotic process is related to polymorphisms. Standard thrombophilia screen tests for heritable thrombophilia generally include factor V Leiden and MTHFR polymorphism tests [12,13,14]. Our patient had homozygous MTHFR (A 1298C) polymorphism and heterozygous factor V Leiden (G1691A) mutation, at least. Association of factor V Leiden with inferior vena cava agenesis (IVCA) has been previously reported [15,16,17]. MTHFR C677T mutation, in homozygous form, causes hyperhomocysteinemia and homocysteinuria that would lead to venous thrombosis four times than normal phenotype [18]. On the other hand, a homozygous FVR2 mutation was shown to cause APC resistance and its heterozygous form when exists with heterozygous factor V Leiden mutation, was shown to increase venous thromboembolism risk 10 times [19]. This genotype combination confirms that patient had increased risk for venous thrombosis and hereditary thrombophilia phenotype.

In a case report, Ramanathan *et al.* presented the first association between perinatal IVC thrombosis and subsequent infrarenal IVC absence or agenesis. This case report is important since it supports the theory of d’Archambeau and Milner that proposes that infrarenal absence of the IVC is not of embryonic origin, thus it is not a real agenesis and is the result of intrauterine or perinatal thrombosis [4]. In fact, there are two theories about the etiology of agenesis/absence of IVC: the first one is congenital absence, or in other words agenesis [2,3] and the second one is early IVC thrombosis in the perinatal period [4,20,21]. The congenital absence theory is problematic for a single embryological event does not fully explain agenesis of IVC [1,4]. The embryology of IVC includes formation, regression and anastomosis of three sets of paired veins (postcardinal, subcardinal, and supracardinal veins) and is a complex process [21].

Although our case was not in the perinatal period, findings, including dilated and prominent superficial veins both in the lower extremity and abdomen and the secondary pulmonary hypertension encountered later suggest that the thrombotic process was in subacute/chronic phase. Furthermore, in the second color Doppler ultrasound examination and CT scan performed six months later we observed that the previously preserved suprarenal segment of the IVC became thrombosed as well. This finding is also suggestive for a chronic and progressive process. Hence, we believe that the present case is a good example for *early IVC thrombosis* theory as the cause of absence of IVC.

In conclusion, this patient had Down syndrome, polymorphism in some selected genes is associated with high risk of venous thrombosis, and some polymorphisms were reported for the first time in literature in an IVCA patient. We hope this would be a pioneering contribution to the etiology of IVC agenesis.

## Competing Interests

The authors declare that they have no competing interests.
